# The Importance of *Microcoleus vaginatus* in Shaping Bacterial Communities Essential for the Development of Cyanobacterial Biological Soil Crusts

**DOI:** 10.3390/microorganisms14030542

**Published:** 2026-02-27

**Authors:** Ziqing Guo, Chunying Wang, Yanfu Ji, Kai Tang, Huiling Guo, Jianyu Meng, Xiang Ji, Shengnan Zhang

**Affiliations:** 1 Inner Mongolia Duolun Hunshandake Sandland Ecosystem Observation and Research Station, Inner Mongolia Academy of Forestry, Hohhot 010010, China; 2 Laboratory for Environmental Microbiology and Biotechnology in Arid and Cold Regions, College of Life Sciences, Inner Mongolia Agricultural University, Hohhot 010018, China; 3 Key Laboratory of State Forestry and Grassland Administration for Sandy Land Biological Resources Conservation and Cultivation, Hohhot 010010, China

**Keywords:** *Microcoleus vaginatus*, Cyano-BSCs, bacteria, community assembly

## Abstract

Biological soil crusts (BSCs) are critical ecological components in arid lands. Their formation and stability hinge on the assembly and interactive networks of cyanobacteria-led bacterial communities. Yet, how different functional cyanobacteria shape the underlying microbial structure and assembly rules is poorly understood. Here, we cultivated artificial algal crusts using two representative cyanobacteria: the nitrogen-fixing *Leptolyngbya* sp. and the non-nitrogen-fixing *Microcoleus vaginatus* (*M. vaginatus* CM01). A total of six treatments were established based on the presence or absence of spraying with in situ BSCs leachate: a control group without inoculation of algae or bacteria (soil, S); a treatment group sprayed only with bacterial suspension (soil + bacteria, SB); a treatment group sprayed only with *M. vaginatus* CM01 (soil + *M. vaginatus* CM01, SM); a treatment group co-inoculated with both BSCs leachate and *M. vaginatus* CM01 (soil + *M. vaginatus* CM01 + bacteria, SMB); a treatment group inoculated only with *Leptolyngbya* sp. CT01 (soil + *Leptolyngbya* sp. CT01, SL); and a treatment group co-inoculated with *Leptolyngbya* sp. CT01 and biocrust leachate (soil + *Leptolyngbya* sp. CT01 + bacteria, SLB). By integrating 16S rRNA gene sequencing, neutral community modeling (NCM), and structural equation modeling (SEM), we dissected differences in Cyano-BSCs development, bacterial community composition, co-occurrence networks, and assembly mechanisms. Inoculation with *M. vaginatus* CM01 (SM, SMB) superiorly promoted Cyano-BSCs development: the SM group achieved the highest coverage (23.33%), while the SMB group showed marked increases in organic matter (OM, 4.10 g·kg^−1^) and chlorophyll *a* (Chl*a*, 13.40 μg·g^−1^), alongside a >5-fold rise in bacterial, cyanobacterial, and nitrogen-fixation gene abundances versus controls. The mechanism centers on extracellular polymeric substances (EPS) secreted by *M. vaginatus*, which homogenized the microenvironment, suppressed stochastic bacterial dispersal (NCM, SM: R^2^ = 0.698), and enhanced deterministic selection. This process forged a highly cooperative network (89.74% positive links, average degree 34.71) that directionally enriched Cyanobacteria (relative abundance 40.40%). The Shannon index of Cyano-BSCs from the group (SMB) reached 7.72 ± 0.09, reflecting high microbial community diversity. SEM confirmed *M. vaginatus* directly regulated bacterial assembly (path coefficient = 0.59, *p* < 0.05) and indirectly improved the soil environment (path coefficient = 0.64, *p* < 0.05), establishing a “cyanobacteria-community-environment” feedback loop. Conversely, the *Leptolyngbya* sp. groups (SL, SLB), despite enriching nitrogen-fixing bacteria and fungi, exhibited low carbon fixation efficiency (notably 1.26 g·kg^−1^ OM in SL) and lack of EPS; communities remained stochastic (NCM, SL: R^2^ = 0.751) with no effective regulatory pathway—a pattern mirrored in S and SB groups. Our findings demonstrate that *M. vaginatus* acts as a core engineer of biological soil Cyano-BSCs formation via an “EPS-mediated habitat filtering—functional group enrichment—cooperative network assembly” cascade, enforcing deterministic community construction. *Leptolyngbya* sp., with limited niche-constructing ability, fails to exert comparable control. This work provides a targeted framework for the artificial restoration of Cyano-BSCs in arid zones.

## 1. Introduction

Desertification is one of the most significant and urgent ecological issues facing the world today [1]. Biological soil crusts (BSCs), as a key component of arid and semi-arid ecosystems, are specialized structures formed by the interaction of bacteria, fungi, cyanobacteria, lichens, mosses, and soil particles [2,3,4]. Due to their resilience to extreme environments such as high temperatures, drought, and intense radiation, BSCs require minimal water for natural growth and are easy to maintain, making them an optimal choice for ecological restoration in desert areas [5]. They play a crucial role in improving soil nutrient conditions, facilitating carbon and nitrogen cycling, promoting soil microbial and vegetation succession, and enhancing biodiversity [2,6].

Cyanobacterial biological soil crusts (Cyano-BSCs) constitute the initial developmental stage of BSCs, a pivotal phase that underpins the formation and subsequent succession of these crusts. Cyanobacteria, as pioneer species in BSCs, not only provide organic carbon sources through photosynthesis but also convert atmospheric free nitrogen into biologically available forms through nitrogen fixation [7]. It is estimated that BSCs contribute approximately 3.6 Pg C of global net carbon uptake and about 107 Tg N of nitrogen fixation annually [8]. The nitrogen fixation process of heterocystous cyanobacteria relies on the nitrogenase system enriched in heterocysts [7,9]. In contrast, non-heterocystous cyanobacteria lack specialized heterocyst structures and do not accomplish nitrogen fixation via nitrogenase in vegetative cells; instead, they primarily achieve this process through a temporal separation mechanism (fixing N_2_ at night) [10]. Furthermore, such cyanobacteria may adopt an alternative pathway, using sulfide: quinone oxidoreductase to perform anoxygenic photosynthesis with sulfide as the electron donor, thereby providing energy for their normal physiological activities and nitrogen fixation processes [11]. Notably, *Leptolyngbya* sp., an important group of Oscillatoriales cyanobacteria, exhibits an extremely wide distribution—it is found not only in BSCs of arid regions such as the Kubuqi Desert, Gurbantunggut Desert, and Mu Us Sandy Land but also in those of semiarid and Mediterranean climate zones in Chile [12,13,14]. As one of the dominant filamentous cyanobacterial groups in these habitats, this species is a non-heterocystous cyanobacterium with the ecological function of biological nitrogen fixation, and its distribution characteristics and ecological roles show high consistency across diverse global habitats. Furthermore, the ecological function of this group in mediating nitrogen cycling has been verified in agricultural habitats: in paddy field ecosystems, *Leptolyngbya* sp. is a major contributor to biological nitrogen fixation in paddy soils developed from purple soil, while it also serves as a dominant genus in the nitrogen-fixing microbial community on maize leaves in acidic red soil [15,16].

Nitrogen-fixing capacity varies significantly among different cyanobacterial groups, and *M. vaginatus* (Oscillatoriales) is a typical species lacking nitrogen-fixation-related genes. As an oligotrophic cyanobacterium adapted to oligotrophic habitats and a core species of BSCs worldwide, it has become a dominant colonizer in low-nitrogen environments by virtue of its dual functions of carbon fixation and direct input of photosynthates into the soil [17,18]. *M. vaginatus* can form stable and nutrient-rich microhabitats by physically binding soil particles through its trichomes and secreting extracellular polymeric substances (EPS), which also constitutes the core theoretical distinction between this species and *Leptolyngbya* sp.—relying solely on its inherent oligotrophic adaptive traits, it secretes EPS to form protective bundle-like structures and releases targeted metabolic signaling molecules, thereby selectively enriching nitrogen-fixing microorganisms such as *Bacillus*, *Arthrobacter* and *Massilia* in its cyanosphere [19,20,21], rather than randomly enriching soil microbes. This further promotes the establishment of a mutualistic symbiotic relationship based on carbon-nitrogen exchange, facilitating its efficient colonization and niche occupation in the soil [21]. This specialized strategy clearly elucidates the colonization mechanism of non-nitrogen-fixing cyanobacteria in nitrogen-limited environments. At present, most relevant studies have focused on the single ecological function of nitrogen-fixing cyanobacteria, while definite conclusions have not yet been formed regarding the ecological contributions of non-nitrogen-fixing cyanobacteria and their synergistic and competitive mechanisms with bacterial communities. As an important component of BSCs living organisms, microorganisms can directly or indirectly participate in all ecological processes of BSCs. However, current research has primarily focused on the singular functions of nitrogen-fixing cyanobacteria, leaving the ecological contributions of non-nitrogen-fixing cyanobacteria and their synergistic and competitive mechanisms with bacterial communities unclear.

Microorganisms are essential components of BSCs and are involved directly or indirectly in all ecological processes of BSCs [22]. Microbial co-occurrence networks can reveal species interaction patterns within communities, while core microorganisms (such as *Sphingomonas*) often serve as functional hubs driving carbon and nitrogen cycles [23]. For instance, in the algal biocrusts of the Gurbantünggüt Desert, the synergistic interactions between cyanobacteria and members of the phyla Proteobacteria and Actinobacteria dominate carbon fixation and nitrate reduction processes [24]. Selecting BSCs microorganisms with specific functions can not only serve as biological indicators to assess environmental changes and ecological risks but can also be used as bioremediators to improve soil quality and enhance ecosystem services, providing scientific basis and technical support for the cultivation of artificial biocrusts and the restoration of damaged ecosystems [25].

The artificial inoculation of cyanobacteria has been widely applied to accelerate Cyano-BSCs formation, primarily using species such as *M. vaginatus*, *Pseudanabaena*, and *Nostoc*. Various inoculation scales have achieved significant results in shortening the time required for Cyano-BSCs formation [26,27]. The development and functionality of Cyano-BSCs depend not only on the primary carbon fixation by cyanobacteria but are also closely related to the interaction networks of microbial communities. However, the effects of nitrogen-fixing and non-nitrogen-fixing cyanobacteria on bacterial co-occurrence networks and the composition of key species in artificial Cyano-BSCs have not been systematically revealed. Additionally, due to the limited diversity of algal species in artificial BSCs, the lack of EPS and microbial communities results in weak resistance to disturbances, making it difficult to meet engineering requirements for wind erosion control in the short term. Therefore, this study focuses on the Cyano-BSCs in the desert region of Inner Mongolia, collecting in situ Cyano-BSCs samples, isolating algal resources, and preparing algal solutions and in situ biocrust leachates to spray on the surface of sandy soil. The aim is to cultivate Cyano-BSCs through in situ inoculation. The research emphasizes the differential impacts of nitrogen-fixing and non-nitrogen-fixing cyanobacteria on the formation and functionality of Cyano-BSCs, with the goal of providing theoretical foundations and technical support for the restoration of desert ecosystems.

## 2. Materials and Methods

### 2.1. Microcosm Experimental Design and Sample Collection

**Figure 1:**
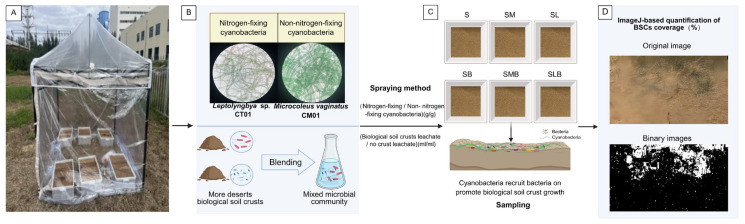
Overview of the experimental design. (**A**) Schematic of the transparent tent field setup; (**B**) Schematic illustrating the inoculation design for the different treatments; (**C**) Depiction of Cyano-BSCs formation across the six treatments; (**D**) Workflow for image-based Cyano-BSCs coverage analysis. Note: S, soil control without inoculation; SB, soil inoculated with biocrust leachate alone; SM, soil inoculated with *M. vaginatus* CM01 alone; SMB, soil co-inoculated with *M. vaginatus* CM01 and biocrust leachate; SL, soil inoculated with *Leptolyngbya* sp. CT01 alone; SLB, soil co-inoculated with *Leptolyngbya* sp. CT01 and biocrust leachate.

In May 2023, samples of BSCs were collected from various desert regions in Inner Mongolia, including the Hunshandak Sandy Land, Horqin Sandy Land, Mu Us Sandy Land, Kubuqi Desert, Ulan Buh Desert, Tengger Desert, Badain Jaran Desert, and Hulunbuir Sandy Land. The specific coordinates of the sampling points are provided in Table S1. The samples were ground using a sterilized mortar and pestle, and sterile water was added to create a biocrust leachate. The leachate was then centrifuged (1000 rpm for 3 min), thoroughly shaken, and filtered through a 0.45 µm membrane to remove algae and other impurities. The filtered BSCs leachate was collected for subsequent use, and a small aliquot was inoculated into BG11 medium. The culture was considered a pure “source strain” only if no green color developed after 14 days of incubation in a shaking flask, indicating the absence of other algae. Additionally, filamentous nitrogen-fixing cyanobacteria (*Leptolyngbya* sp.) and non-nitrogen-fixing cyanobacteria (*M. vaginatus*) isolated from the aforementioned desert regions were identified as the target species via combined morphological observation and molecular phylogenetic analysis. Sandy soil was collected from the northern Kubuqi Desert on the Ordos Plateau (40°07′59.5820″ N, 111°00′19.8028″ E) and subjected to three rounds of intermittent sterilization. The soil was then placed in plastic culture boxes measuring approximately 20 cm × 30 cm × 13 cm. For the treatments with algal mud and BSCs leachate, 1 g of algal mud (prepared from a single batch to ensure experimental consistency, with cyanobacterial cells harvested at the exponential growth phase by centrifugation at 1400× *g* for 5 min) was applied to the surface of sandy soil together with 100 mL of BSCs leachate. For the other treatments without BSCs leachate, the soil was rehydrated and sprayed with 100 mL of BG11 medium instead. The target cyanobacteria in this study are filamentous and prone to entanglement and fragmentation, which renders precise quantification via cell counting unfeasible. Thus, algal mud from the same batch was used for inoculation at a wet weight of 1 g to ensure uniform cyanobacterial inoculum levels across all treatments—a method widely adopted in artificial BSCs cultivation [26,28,29]. The experiment was conducted under a transparent tent in an open outdoor area to exclude precipitation and aeolian deposition (Figure 1A). Six treatments were established, each with five replicates (experimental design shown in Figure 1B): a non-inoculated control (soil only, S); soil inoculated with biocrust leachate alone (SB); soil inoculated with *M. vaginatus* CM01 algal mud alone (SM); soil co-inoculated with *M. vaginatus* CM01 algal mud and biocrust leachate (SMB); soil inoculated with *Leptolyngbya* sp. CT01 algal mud alone (SL); and soil co-inoculated with *Leptolyngbya* sp. CT01 algal mud and biocrust leachate (SLB).

Every day at 9:00 AM, 200 mL of sterile water was sprayed, and every seven days, 200 mL of BG11 nutrient solution was added, with no additional water added on the day of nutrient solution application. The cultivation period for the Cyano-BSCs was 30 days (from mid-September to mid-October 2024) (Figure 1C). The coverage of Cyano-BSCs was analyzed using ImageJ software (version 1.53f): after capturing color images of Cyano-BSCs growth, we selected the green band (which maximizes the distinction between Cyano-BSCs and the soil background) and applied a validated threshold (grayscale values 85–255) to convert the images to binary format. The particle analysis module was used to calculate the percentage area of the white regions (representing the covered parts of Cyano-BSCs), and a custom macro was written for batch processing of all images (Figure 1D) [30]. Following this, five different sampling points (1 cm × 1 cm) were selected within each dark patch formed in the treatment, and surface samples were collected from the top 0–2 mm. To ensure the reliability and reproducibility of the experimental results, five independent biological replicates were established for each treatment. For each biological replicate, the samples from the five sampling points were thoroughly homogenized to prepare one composite sample. A total of 6 treatment groups were included in the experiment, yielding 30 composite samples (6 treatments × 5 biological replicates) for subsequent analysis. Additionally, equal amounts of surface soil were collected from the SB treatment group, which did not receive algal solution spraying.

### 2.2. Sample Collection and Soil Property Analysis

Immediately after sampling, visual observations and physicochemical analyses were conducted. DNA analysis samples were preserved at −80 °C until further processing. Chlorophyll *a* (Chl*a*) was extracted using ethanol and measured spectrophotometrically at a wavelength of 665 nm, with centrifugation conditions set at 4000 rpm for 10 min [31]. Soil organic matter (OM) was determined using the K_2_Cr_2_O_7_-H_2_SO_4_ dilution oxidation method, with a reaction time of 30 min at room temperature, and the endpoint was titrated using o-phenanthroline as an indicator [32]. Each composite sample was subjected to five technical replicates.

### 2.3. DNA Extraction, PCR Amplification, and Sequencing

DNA was extracted from 0.5 g of fresh soil from each sample utilizing the FastDNA SPIN, Kit (MP Biomedicals, Irvine, CA, USA). Quality control was performed using a NanoDrop NC2000 spectrophotometer (OD_260/280_ ≥ 1.8; OD_260/230_ ≥ 2.0) and 1% agarose gel electrophoresis (main band > 15 kb), with samples stored at −20 °C. The V3–V4 region of the bacterial 16S rRNA gene was amplified using the 338F primer (ACTCCTACGGGAGGCAGCAG) and the 806R primer (GGACTACHVGGGTWTCTAAT) via the ABI GeneAmp^®^ PCR System 9700 (ABI, Loma Linda, CA, USA). DNA concentration and purity were evaluated using a NanoDrop^®^ ND-2000 spectrophotometer (Thermo Scientific Inc., Waltham, MA, USA). The PCR reaction mixture (25 μL) included 5 μL of 5× Buffer, 0.25 μL of Fast pfu DNA polymerase, 2 μL of 2.5 mM dNTPs, 1 μL of each 10 μM primer, and 1 μL of DNA template. The amplification program consisted of an initial denaturation at 98 °C for 5 min, followed by 25 cycles of denaturation at 98 °C for 30 s, annealing at 53 °C for 30 s, and extension at 72 °C for 45 s, concluding with a final extension at 72 °C for 5 min. The amplicons were purified using VAHTSTM DNA Clean Beads, quantified with Quant-iT PicoGreen, and equimolar mixtures were sequenced on the Illumina NovaSeq platform (NovaSeq 6000 SP kit, 500 cycles) (Shanghai Pasono Biotechnology, Shanghai, China).

Raw sequencing data underwent quality control using Fastp (v1.0) [33], and sequences were assembled using FLASH (v1.2.11) [34]. DADA2 (v2022.11) was utilized with default parameters to denoise the quality-controlled sequences, excluding chloroplast and mitochondrial sequences, and all samples were rarefied to 20,000 sequences (Good’s coverage > 99.9%). Species annotation was conducted using the QIIME2 (v2022.11) naive Bayes classifier based on the Silva 138 (16S rRNA) databases [35]. Alpha diversity (Shannon) and beta diversity (Bray–Curtis) were calculated following the methods outlined in reference [36].

### 2.4. Quantification of Gene Copy Numbers

Using primers referenced in the literature [37], absolute quantification of bacterial 16S rRNA, fungal 18S rRNA, cyanobacterial 16S rRNA, nitrogen-fixing microbial *nif*H genes was performed on the LightCycler 480II system. The reaction mixture (20 μL) contained 10 μL of SYBR^®^ Premix Ex Taq (TaKaRa, San Jose, CA, USA), 0.5 μM of each primer pair, and template DNA (5–10 ng). Each sample was analyzed in triplicate. Amplification specificity was verified through 1.5% agarose gel electrophoresis (single target band) and a single peak melting curve. Gene copy numbers were normalized to per gram of dry soil weight. Primers were synthesized by Shanghai Sangon Biotech, Shanghai, China.

### 2.5. Statistical Analysis

To investigate the alterations in bacterial community co-occurrence patterns across different treatments, we performed a network analysis based on Spearman correlation for Cyano-BSCs soil samples. To reduce the likelihood of false correlations stemming from rare taxa, we concentrated on the most abundant bacterial phylotypes, specifically the top 300 ASVs. Although these phylotypes constitute a minority within the overall community, they are anticipated to play crucial roles in the ecosystem. The network was constructed using established stringent correlation thresholds, specifically an absolute Spearman correlation coefficient (*p*) greater than 0.6 and a false discovery rate (FDR) adjusted *p* value of less than 0.001 [38,39]. Visualization of the network was achieved using Gephi software (version 0.9.2) with the Fruchterman-Reingold layout algorithm [40]. Node topological features, including degree, betweenness, closeness, and eigenvector centrality, were calculated using the “igraph” package (v1.0.0).

To evaluate the topological roles of bacterial species within the network, we calculated the within-module connectivity (Zi) and among-module connectivity (Pi) of each node. According to corresponding criteria, we identified module hubs (Zi > 2.5 and Pi < 0.62), connectors (Zi < 2.5 and Pi > 0.62), network hubs (Zi > 2.5 and Pi > 0.62), and peripherals (Zi < 2.5 and Pi < 0.62). Module hubs, connectors, and network hubs were identified as keystone nodes [41].

To investigate the roles of randomness and determinism in community assembly, we applied a neutral community model (NCM) following the approach of reference [42]. Grounded in neutral theory, the model predicts the relationship between ASV occurrence frequency and their relative abundance [43]. We executed 1000 bootstrap repetitions to compute the 95% confidence intervals for all fitted statistics. Subsequently, ASVs were categorized into upper, lower, and neutral partitions based on whether their occurrence frequency fell above, below, or within the 95% confidence interval. The module explanatory power (Rsqr, R^2^) was calculated to assess the overall model fit, with the parameter “Nm” indicating the extent of migration diffusion.

Structural equation modeling (SEM) was developed to evaluate the potential mechanisms through which soil physicochemical properties (OM, coverage) and key nodes influence bacterial community structure under various algal species applications. This was implemented using IBM SPSS AMOS (version 23.0) with maximum likelihood estimation. Model fit was assessed using several indicators: chi-square test (χ^2^, *p* > 0.05), comparative fit index (CFI > 0.90), and root mean square error of approximation (RMSEA < 0.05). Lower χ^2^ values (*p* > 0.05), higher CFI values (>0.90), and lower RMSEA values (<0.05) indicate a good fit between the model and the data. Additionally, a smaller Akaike Information Criterion (AIC) value suggests a more parsimonious model with superior fit. In the SEM analysis, Cyano-BSCs growth indicators (OM, coverage) were integrated through principal component analysis (PCA), and the first principal component (PC1) score was extracted as a representative indicator of the overall environmental gradient. Keystone taxa were defined as ASVs positioned in the connector category on the co-occurrence network Zi-Pi plot, with their abundance data reduced in dimensionality through PCoA, extracting the PCo1 score as a representative indicator of keystone taxa. The microbial community structure was characterized by the PCo1 axis score derived from PCoA analysis of the ASV composition matrix [44].

To assess differences among treatment groups, soil physicochemical parameters and functional gene abundance data were statistically analyzed using SPSS software (version 26.0). The quantitative PCR (qPCR) data for functional genes were presented in box plots, where the box represents the interquartile range (IQR) and the line within the box indicates the median. All data underwent Shapiro–Wilk normality tests prior to analysis; data meeting the normal distribution criteria (*p* > 0.05) were analyzed using one-way ANOVA. If the ANOVA results were significant, Duncan’s multiple range test was performed for post hoc analysis (*α* = 0.05). Significant differences between groups were denoted in the figures using letter notation, with a significance threshold set at *p* < 0.05.

## 3. Results

### 3.1. Growth Status of Cyano-BSCs Under Different Treatments

Inoculation with *M. vaginatus* significantly promoted the formation of Cyano-BSCs and increased the surface organic matter (OM) and Chl*a* content. No Cyano-BSCs formation was observed in the treatments without algal inoculation (S, SB). Inoculation with *Leptolyngbya* sp. resulted in Cyano-BSCs formation, but the coverage was low (3.10%). After 30 days of cultivation, both the SM and SMB treatment groups demonstrated significantly higher Cyano-BSCs coverage and OM content compared to other treatments (*p* < 0.05). The SM treatment achieved the highest Cyano-BSCs coverage of 23.33%. The highest OM content (4.10 g·kg^−1^) was recorded in the SMB treatment, representing an increase of 22.02%, 225.40%, and 95.24% compared to the SM, SL, and SLB treatments, respectively. A consistent trend was observed for Chl*a* concentration, with the SMB group showing a significantly higher value (13.40 μg·g^−1^) than all other treatments (*p* < 0.05) (Table 1).

### 3.2. Abundance of Bacterial, Fungal, Cyanobacterial, and Nitrogen-Fixing Genes

**Figure 2:**
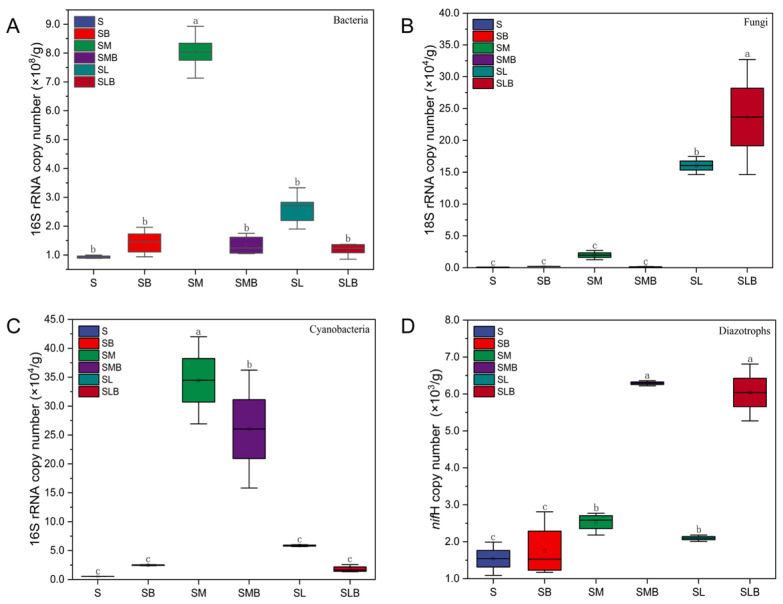
Copy numbers per gram of sample for bacterial (**A**), fungal (**B**), cyanobacterial (**C**), and nitrogen-fixation (**D**) genes in Cyano-BSCs under different treatments. Data are presented as mean ± SE. Different lowercase letters (a, b, c) denote significant differences (Duncan’s multiple range test; *p* < 0.05). note: S, soil control without inoculation; SB, soil inoculated with biocrust leachate alone; SM, soil inoculated with *M. vaginatus* CM01 alone; SMB, soil co-inoculated with *M. vaginatus* CM01 and biocrust leachate; SL, soil inoculated with *Leptolyngbya* sp. CT01 alone; SLB, soil co-inoculated with *Leptolyngbya* sp. CT01 and biocrust leachate. Values are presented as mean ± standard deviation (*n* = 5).

The copy numbers of the bacterial and cyanobacterial 16S rRNA genes, as well as the *nif*H gene of diazotrophs, were significantly higher in treatments inoculated with *M. vaginatus* (SM, SMB) compared to all other treatments (*p* < 0.05; Figure 2). Specifically, the abundance of the *nif*H gene in the SMB treatment was comparable to that in the SLB treatment and significantly exceeded the levels in the SM, SL, S, and SB treatments by factors of 2.49, 3.00, 4.21, and 4.60, respectively. The single bacteria inoculation treatment (SB) exhibited low copy numbers for the 16S rRNA, 18S rRNA, and *nif*H genes, with its total bacterial abundance (16S rRNA) being substantially lower than that in the SM and ST treatments. The cyanobacterial 16S rRNA gene copy numbers in the *M. vaginatus* treatments (SM, SMB) were significantly higher, by at least fivefold, than those in the SL and SLB treatments. In contrast, the *Leptolyngbya* sp. treatment (SL) demonstrated a specific enrichment effect on fungi and diazotrophs. Its fungal 18S rRNA gene copy number (1.61 × 10^5^/g) was 78.2 and 7.1 times greater than that in the SB and SM groups, respectively (*p* < 0.05). Concurrently, the abundance of the diazotroph *nif*H gene increased significantly to 2.10 × 10^3^/g in the SL treatment compared to the SB group (*p* < 0.05). Overall, with the exception of lower fungal abundance, the *M. vaginatus* inoculations (SM, SMB) yielded higher abundances of bacteria, cyanobacteria, and nitrogen-fixation genes than other treatments, indicating the potential of *M. vaginatus* inoculation to enhance the absolute abundance of these three microbial groups.

### 3.3. Bacterial Community Composition and Diversity in Cyano-BSCs

**Figure 3:**
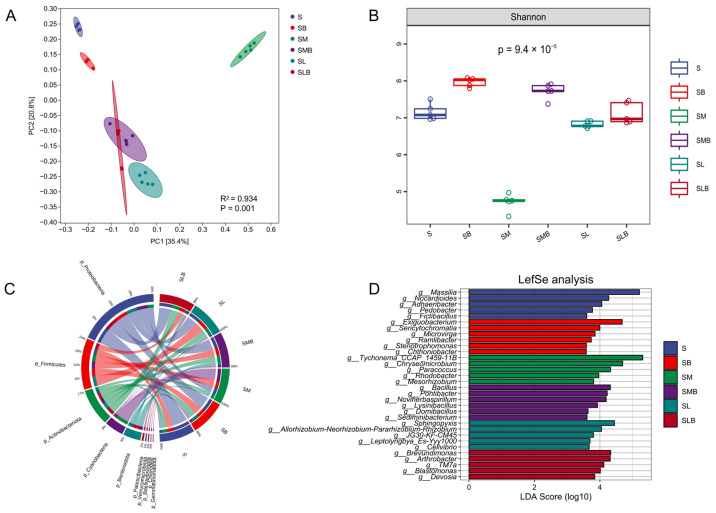
Microbial community structure and diversity of Cyano-BSCs under different treatment conditions. (**A**) Principal coordinate analysis (PCoA) based on Bray–Curtis distances; each point represents one sample (5 replicates per group), different colored points indicate the above groups, with 95% confidence ellipses for group distinction. (**B**) Alpha diversity index (Shannon); (**C**) Bacterial community composition at the phylum level; (**D**) Distribution of LDA scores for differentially abundant taxa. Note: S, soil control without inoculation; SB, soil inoculated with biocrust leachate alone; SM, soil inoculated with *M. vaginatus* CM01 alone; SMB, soil co-inoculated with *M. vaginatus* CM01 and biocrust leachate; SL, soil inoculated with *Leptolyngbya* sp. CT01 alone; SLB, soil co-inoculated with *Leptolyngbya* sp. CT01 and biocrust leachate. Values are presented as mean ± standard deviation (*n* = 5).

To further elucidate the impact of different treatments on Cyano-BSCs formation, bacterial 16S rRNA gene amplicon sequencing was employed to analyze the compositional differences, abundance dynamics, and community assembly characteristics of the soil microbiome under treatments involving single cyanobacterial inoculation (SM, SL), single microbial inoculation (SB), and cyanobacterial-microbial co-inoculation combinations (SMB, SLB). Bray–Curtis-based PCoA revealed significant differences in bacterial composition among the inoculation treatments (*p* = 0.001, PERMANOVA with Adonis test), with distinct separation of treatment groups along PC1 (explaining 35.4% of variance) and PC2 (explaining 20.8% of variance) axes (Figure 3A). The Shannon index differed significantly among the single bacterial inoculation (SB: 7.90 ± 0.05), *M. vaginatus* CM01 (SM: 4.85 ± 0.10), and *Leptolyngbya* sp. CT01 (SL: 6.82 ± 0.04) treatments (*p* < 0.05). This indicates that cyanobacterial inoculation significantly alters sandy soil surface microbial diversity, with SB (single bacteria) showing significantly higher diversity than the cyanobacteria-only treatments (SM, SL). Meanwhile, bacteria-*M. vaginatus* co-inoculation (SMB: 7.72 ± 0.09) significantly increased the Shannon index of formed Cyano-BSCs (Figure 3B), which was higher than that of the two *Leptolyngbya* sp.-inoculated treatments (SL: 6.82 ± 0.04, SLB: 7.12 ± 0.13). Analysis at the phylum level across treatments identified ten major bacterial phyla (Figure 3C), with nine dominant phyla (collective relative abundance > 99%) governing the community structure: Proteobacteria, Firmicutes, Actinobacteriota, Cyanobacteria, Bacteroidota, Patescibacteria, Verrucomicrobiota, Chloroflexi, and Gemmatimonadota. As shown in Figure 3D, treatments inoculated with *M. vaginatus* (SM, SMB) were primarily enriched for bacterial groups such as *Rhodobacter*, *Mesorhizobium*, *Paracoccus*, *Bacillus* and *Lysinibacillus*.

### 3.4. Co-Occurrence Network Patterns of Bacteria in Cyano-BSCs

**Figure 4:**
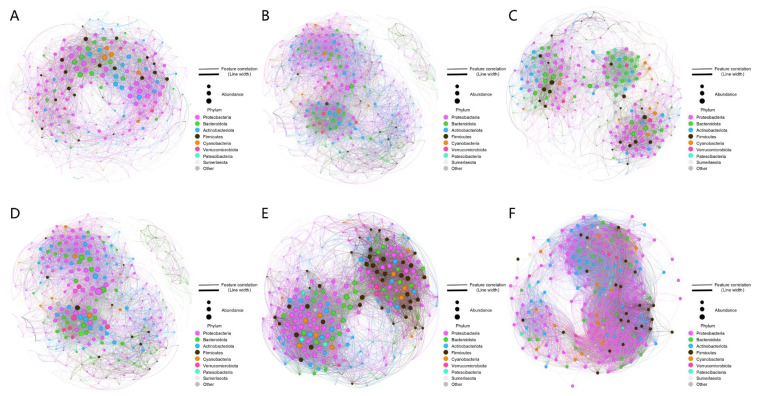
Co-occurrence networks of bacterial ASVs: (**A**) Control without algal or bacterial inoculation (soil, S); (**B**) Treatment with bacterial suspension only (soil + bacteria, SB); (**C**) Treatment with *M. vaginatus* only (soil + *M. vaginatus* CM01, SM); (**D**) Treatment co-inoculated with Cyano-BSCs extract and *M. vaginatus* (soil + *M. vaginatus* CM01 + bacteria, SMB); (**E**) Treatment with *Leptolyngbya* sp. only (soil + *Leptolyngbya* sp. CT01, SL); (**F**) Treatment co-inoculated with Cyano-BSCs extract and *Leptolyngbya* sp. (soil + *Leptolyngbya* sp. CT01 + bacteria, SLB). Values are presented as mean ± standard deviation (*n* = 5).

Network complexity analysis confirmed the microbial recruitment advantage conferred by inoculation with *M. vaginatus* (SM and SMB) (Table 2). The control group (S) comprised only 263 nodes and 3698 edges. In contrast, the SM treatment exhibited a significantly higher number of nodes (319) and edges (5536) not only compared to the control but also relative to the single *Leptolyngbya* sp. inoculation (SL: nodes = 312; edges = 4127). Notably, the SM group had the highest proportion of positive connections (89.74%) among all groups, with an average degree (avgK) of 34.71 and a network density (D) of 0.109, indicating the optimal proportion of positive associations among microorganisms in this group, along with strong interspecific connectivity and community recruitment capacity. In contrast, the synergistic inoculation treatment (SMB) achieved a multiplicative enhancement of network structural advantages, with its number of edges (8497), average clustering coefficient (avgCC = 0.658), and network density (D = 0.239) all significantly increased, forming a highly aggregated microbial co-occurrence network. Co-inoculation (SMB) amplified these advantages, leading to significant increases in edge count (8497), average clustering coefficient (avgCC = 0.66), and network density (D = 0.24), thereby forming a highly aggregated network. By comparison, the pure microbial consortium treatment (SB) only showed prominence in modularity (RM = 0.16, *p* < 0.05), while the advantages of the ST and STB treatments were substantially lower than those of SM and SMB. Although SLB presented a slightly higher edge count (8999 vs. 8497 of SMB) and a comparable average degree (63.24 vs. 63.60 of SMB), the emphasis on “greater connectivity” for the SMB network refers specifically to functional connectivity rather than structural metrics alone. The SMB network maintained a higher positive association ratio relative to SLB, a metric directly indicative of interspecific cooperation and mutualism that is critical for driving the functional synergy required for Cyano-BSCs development. By contrast, structural metrics (edge count and avgK) do not distinguish between cooperative (positive) and competitive (negative) interactions, and thus are less informative for evaluating the network’s ecological relevance to Cyano-BSCs formation. ASV co-occurrence diagrams (Figure 4A–F) visually confirmed these patterns: the S network was sparse, the SL network complexity was limited, whereas the SM network was dense, and the SMB network exhibited even greater connectivity, clearly demonstrating the potent recruitment effect of *M. vaginatus*. This “highly synergistic and aggregated” network provides crucial support for stable Cyano-BSCs growth by strengthening functional complementarity and resource utilization. In summary, inoculation with *M. vaginatus* (SM, SMB) significantly enhanced microbial recruitment capacity by increasing network complexity and positive interactions, establishing a network structure conducive to Cyano-BSCs development through the dual effects of “*M. vaginatus* induction” and “microbial synergy”.

**Figure 5:**
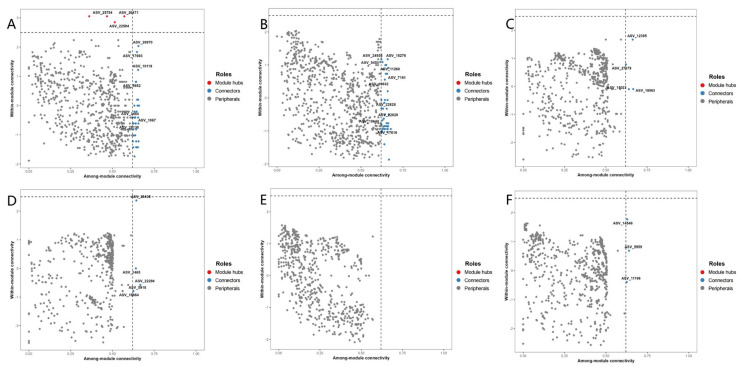
Identification of keystone bacterial ASV taxa. (**A**) Control without algal or bacterial inoculation (soil, S); (**B**) Treatment with bacterial suspension only (soil + bacteria, SB); (**C**) Treatment with *M. vaginatus* only (soil + *M. vaginatus* CM01, SM); (**D**) Treatment co-inoculated with Cyano-BSCs extract and *M. vaginatus* (soil + *M. vaginatus* CM01 + bacteria, SMB); (**E**) Treatment with *Leptolyngbya* sp. only (soil + *Leptolyngbya* sp. CT01, SL); (**F**) Treatment co-inoculated with Cyano-BSCs extract and *Leptolyngbya* sp. (soil + *Leptolyngbya* sp. CT01 + bacteria, SLB). Values are presented as mean ± standard deviation (*n* = 5).

Based on Zi-Pi plots (Figure 5A–F), keystone species were identified. No network hubs were found across all treatments. However, significant differences in keystone species characteristics were observed. Only the non-inoculated control (S) contained module hubs (ASV_25754/26471/22594), belonging to the phyla Firmicutes and Proteobacteria. The keystone species in the SB, SM, SL, and SLB treatments were all connectors, with varying phylum-level compositions: the SB group included Bacteroidota, Gemmatimonadota, and Proteobacteria; the SM group was predominantly Proteobacteria, accompanied by Bacteroidota and Sumerlaeota; the SL group involved Proteobacteria, Bdellovibrionota, and Actinobacteriota; and the SLB group was primarily concentrated in Proteobacteria. Notably, the co-occurrence networks of treatments inoculated with *M. vaginatus* (SM, SMB) (Figure 4B,E) visually demonstrated that nodes from the phyla Cyanobacteria and Proteobacteria consistently exhibited larger size and denser connections. This pattern directly reflects the positive correlation between species abundance (node size) and connectivity, with high-abundance Proteobacteria taxa showing particularly prominent connectivity. As core connector species, these bacterial groups likely enhance community diversity through functional complementarity.

### 3.5. Mechanisms of Bacterial Community Assembly Driven by Cyanobacterial Functional Differentiation

**Figure 6:**
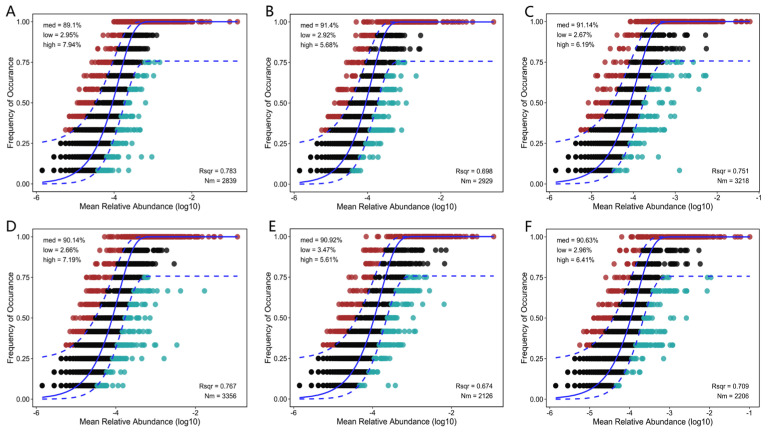
Neutral community model (NCM) fitting results for microbial community assembly under different treatments: (**A**) Control without algal or bacterial inoculation (soil, S); (**B**) Treatment with bacterial suspension only (soil + bacteria, SB); (**C**) Treatment with *M. vaginatus* only (soil + *M. vaginatus* CM01, SM); (**D**) Treatment co-inoculated with Cyano-BSCs extract and *M. vaginatus* (soil + *M. vaginatus* CM01 + bacteria, SMB); (**E**) Treatment with *Leptolyngbya* sp. only (soil + *Leptolyngbya* sp. CT01, SL); (**F**) Treatment co-inoculated with Cyano-BSCs extract and *Leptolyngbya* sp. (soil + *Leptolyngbya* sp. CT01 + bacteria, SLB). The solid blue line indicates the best-fit curve of the NCM, and the dashed blue lines represent the 95% confidence intervals predicted by the model. The solid blue line represents the best-fit line of the model, and the dashed blue line denotes its 95% confidence interval for model predictions. Red dots indicate ASVs with observed occurrence frequencies higher than the model predictions, green dots represent those with frequencies lower than the predictions, and black dots signify ASVs with frequencies falling within the predicted range. Values are presented as mean ± standard deviation (*n* = 5).

The sloan neutral community model (NCM) was applied to quantify the relative contributions of stochastic diffusion and deterministic selection during the assembly of bacterial communities within the Cyano-BSCs. A high R^2^ value corresponds to dominance by stochastic processes (random diffusion), whereas a low R^2^ value reflects the influence of deterministic selection, such as microbial habitat filtering or interspecies interactions (Figure 6). The results revealed that the non-inoculated control (S) exhibited the highest R^2^ (0.783), followed by the bacterial inoculum control (SB, R^2^ = 0.767; *p* < 0.05), indicating that stochastic processes were the dominant drivers in these treatments. Following cyanobacterial inoculation, the contribution of neutral processes generally decreased. Single inoculation and co-inoculation groups showed consistent trends: the nitrogen-fixing *Leptolyngbya* sp. single inoculation group (SL, R^2^ = 0.751) and its co-inoculation group (SLB, R^2^ = 0.709) still exhibited a dominance of stochastic processes. In contrast, the non-nitrogen-fixing *M. vaginatus* CM01 single inoculation group (SM, R^2^ = 0.698) showed a further reduction in its co-inoculation group (SMB, R^2^ = 0.674). The SMB group also displayed the lowest migration rate (Nm = 2.126 × 10^3^), indicating a significant reinforcement of deterministic selection.

This finding aligns with the conclusions from the preceding co-occurrence network analysis. *M. vaginatus* likely secretes EPS that homogenize the microhabitat, thereby suppressing random bacterial diffusion (manifested as low R^2^ and Nm values) while strengthening the regulatory role of deterministic selection in community assembly. Conversely, *Leptolyngbya* sp. appears to lack this microhabitat-modulating capability, resulting in the relative contribution of stochastic processes remaining at a comparatively high level (Section 3.4 for details).

**Figure 7:**
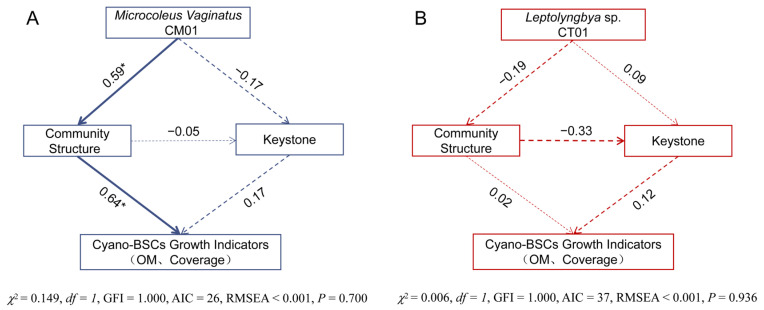
Structural equation models (SEM) of Cyano-BSCs growth indicators, keystone species, and microbial community under different cyanobacterial inoculation treatments. (**A**) *M. vaginatus* inoculation group (treatments SM and SMB); (**B**) *Leptolyngbya* sp. inoculation group (treatments SL and SLB). In the figure, “Cyano-BSCs Growth Indicators” represents the PC1 scores derived from PCA dimensionality reduction of organic matter (OM) and Cyano-BSCs coverage. “Keystone” denotes the PC1 coordinate values from PCoA performed on keystone ASVs located within the Connectors region for the *M. vaginatus* treatments (Figure 5B,C) and the *Leptolyngbya* sp. treatments (Figure 5E,F), respectively. “Community structure” refers to the PC1 coordinate values from PCoA based on microbial community composition data. Path coefficient strength is indicated by arrow width (with values labeled adjacent to arrows). Solid arrows represent positive effects, and dashed arrows represent negative effects; * = *p* < 0.05.

To elucidate the intrinsic mechanisms by which cyanobacteria promote Cyano-BSCs development, structural equation modeling (SEM) was employed to analyze the effects of cyanobacterial species, microbial community structure, and keystone species on core indicators of Cyano-BSCs development (coverage and OM) (Figure 7). The results revealed that inoculation with *M. vaginatus* exerted a significant positive driving effect on the restructuring of the microbial community (path coefficient = 0.59, *p* < 0.05). The restructured microbial community, in turn, directly regulated environmental and growth factors associated with Cyano-BSCs development (path coefficient = 0.64, *p* < 0.05). This indicates that *M. vaginatus* does not directly influence the Cyano-BSCs formation process. Instead, it indirectly enhances Cyano-BSCs coverage and OM accumulation by driving structural reorganization and taxa selection within the microbial community. The synergistic interaction between cyanobacterial inoculation and microbial community restructuring constitutes the core mechanism promoting Cyano-BSCs development in this treatment. In stark contrast, no significant associative paths were detected among cyanobacterial inoculation, microbial community, and Cyano-BSCs development in the *Leptolyngbya* sp. inoculation treatment. This highlights the decisive influence of cyanobacterial species-specific traits on the regulatory pathways governing Cyano-BSCs formation.

## 4. Discussion

### 4.1. The Role of M. vaginatus in Shaping Community Structure

Desert cyanobacteria serve as a core ecological engine in the development of BSCs in arid and semi-arid ecosystems, exhibiting high diversity within BSCs [45,46]. They are among the first colonizers of bare sand habitats due to their unique physiological metabolism and microhabitat modification capabilities [3]. During the colonization process, they continuously secrete EPS, which improve the microhabitat through physical binding, moisture retention, and nutrient retention [47,48,49]. This enhancement promotes the formation of aggregates within the biocrust [50]. Such improvements reduce the intensity of abiotic stress and increase the importance of biotic interactions among microorganisms (cooperation and competition), creating conditions conducive to the colonization of more diverse microbial groups and subsequent succession by lichens and mosses. This drives BSCs towards a more complex structure and a more stable function dominated by lichens and mosses [51,52,53].

*M. vaginatus* mediates carbon (C) and nitrogen (N) exchange with associated cyanosphere heterotrophs [54], forming a specialized nitrogen-fixing microbial niche around itself via the heterotrophs’ nitrogen-fixing function. In this study, the treatment inoculated with *M. vaginatus* significantly enriched various nitrogen-fixing genera, including *Mesorhizobium*, *Lysinibacillus*, and *Rhodobacter*. Previous studies have confirmed the presence of distinct nitrogen-fixing strains within these genera (e.g., *Mesorhizobium atlanticum* sp. nov [55], *Lysinibacillus sphaericus* [56], *Rhodobacter capsulatus* [57]), and their relative abundances in the inoculated treatment were significantly higher than those in the control treatment (*p* < 0.05). This further confirms that the microhabitat associated with *M. vaginatus* has the capacity to recruit and enrich nitrogen-fixing microorganisms. Additionally, several genera known to secrete EPS, such as *Paracoccus* [58] and *Bacillus* [59], were also detected in this treatment, indicating the coexistence of nitrogen-fixing and EPS-producing functional groups within the associated microbial community. Notably, some strains of *Mesorhizobium* (e.g., *Mesorhizobium loti*) possess dual functions: their nitrogen-fixing ability has been confirmed [60], and they are also efficient EPS producers. Under conditions with sucrose as a carbon source and pH 5.5, EPS production by *M. loti* strains SEMIA806 and SEMIA816 can reach 0.33–2.04 g·L^−1^ [61]. These results suggest that *M. vaginatus* may enhance the recruitment of microorganisms with both nitrogen-fixing and EPS synthesis capabilities through selective effects on the associated microenvironment. The functional complementarity of these microorganisms can provide structural support (mediated by EPS) and nutrient supply (through nitrogen fixation) for the formation of microbial Cyano-BSCs, thereby strengthening the ecological functions of the Cyano-BSCs.

The restructuring of the microbial interaction network is another core pathway through which *M. vaginatus* regulates Cyano-BSCs development. In the SM and SMB groups, the copy number of cyanobacterial 16S rRNA gene was at least fivefold higher than that in the SL and SLB groups, with the relative abundance of Cyanobacteria reaching 40.40% at the phylum level, which was consistent with the structure of dominant microbial communities observed in the natural biocrusts of the Gurbantünggüt Desert [62]. Meanwhile, in this study, the Shannon index of Cyano-BSCs formed by the bacteria—*M. vaginatus* co-inoculation group (SMB: 7.72 ± 0.09) was significantly elevated (Figure 3B), and this index was higher than that in the *Leptolyngbya* sp.-inoculated groups. These results confirm that artificial inoculation of *M. vaginatus* can effectively simulate the assembly pattern of microbial communities in natural biocrusts. Furthermore, this community construction is not merely a simple enrichment of species but rather a reinforcement of cooperative functions through the restructuring of the microbial interaction network. Co-occurrence network analysis of bacteria in the SM group revealed 319 nodes and 5536 edges, with a positive connection ratio as high as 89.74%. The average degree (34.71) and network density (0.11) were the highest among single inoculation groups, reflecting a high level of cooperation that corresponds with the stability characteristics of microbial networks in moss biocrusts reported in the Tengger Desert [63]. When *M. vaginatus* was co-inoculated with indigenous microorganisms (SMB), the network’s clustering coefficient (0.66) peaked, and the density (0.24) visually demonstrated the “cooperative recruitment” effect generated by the core species and surrounding microbial communities, thereby creating favorable microecological conditions for Cyano-BSCs development. It is worth noting that although *M. vaginatus*, as a pioneer species in BSCs, does not possess nitrogen-fixing capabilities, it can recruit nitrogen-fixing bacteria to form “mutualistic symbionts” through a GABA/Glu signal-mediated carbon-nitrogen exchange mechanism, thereby enhancing its colonization ability [64]. Neutral models further revealed its community construction mechanism: the lower R^2^ values after inoculation with *M. vaginatus* (SM: R^2^ = 0.698; SMB: R^2^ = 0.674) and lower migration diffusion rates (SM: Nm = 2.93 × 10^3^; SMB: Nm = 2.13 × 10^3^) confirmed that its EPS-mediated microhabitat homogenization inhibited random community diffusion. This physical barrier effect reinforced deterministic selection of indigenous microbial communities, thereby promoting directional succession of microbial community structure. This clearly indicates that the regulation of microorganisms by *M. vaginatus* is not limited to the local shaping of associated microorganisms but is more fundamentally about the directional construction of functional microbial groups—this optimization at the functional level is the core biological mechanism driving Cyano-BSCs development.

### 4.2. M. vaginatus Supported Higher Contents of Organic Matter and Biomass and Its Potential Functions in Cyano-BSCs Formation

This study observed, through a 30-day cultivation experiment, that the *M. vaginatus* inoculation groups (SM, SMB) exhibited significantly higher Cyano-BSCs coverage (up to 23.33%), OM content (up to 4.10 g·kg^−1^), and Chl*a* content (up to 13.40 μg·g^−1^) compared to the *Leptolyngbya* sp. groups (SL, SLB) and the non-inoculated and algal treatment groups (S, SB, *p* < 0.05). In natural ecosystems, the dominance of nitrogen-fixing and non-nitrogen-fixing organisms is influenced by nitrogen availability, with nitrogen-fixing organisms occupying a greater advantage in ecosystems with low nitrogen availability [65]. Although this result appears to contradict the conventional view that nitrogen-fixing cyanobacteria can supplement nitrogen and thus promote the development of BSCs, it actually reveals the core driving mechanism underlying the formation of Cyano-BSCs—a mechanism that transcends the simple nitrogen supply capacity of cyanobacteria, the deterministic assembly ability of bacterial communities dominated by cyanobacteria is the key determinant of BSCs development efficiency. The difference in this core ability between *Leptolyngbya* sp. and *M. vaginatus* directly leads to the divergence in BSCs formation effects. Previous studies have confirmed that some *Leptolyngbya* species (e.g., *Leptolyngbya boryana*) can activate the transcription of *nif* genes through CnfR protein under microaerobic conditions to increase nitrogenase activity, suggesting that *Leptolyngbya* sp. CT01 in this study may also have similar nitrogen-fixing potential [66]. It is worth noting that in the study of arsenic-contaminated soil remediation, the BSCs formed by the co-inoculation of *Leptolyngbya* sp. XZMQ and plant growth-promoting bacteria can effectively improve soil water retention, soil structure, enzyme activity, and thus soil fertility, with significant remediation effects on arsenic pollution [67]. However, this advantage was not reflected in the arid desert BSCs cultivation scenario of this study, further confirming the key influence of habitat specificity on the functional performance of cyanobacteria.

Scanning electron microscope observations have confirmed that the filamentous sheath structure of *M. vaginatus* can form mechanical constraints on sand particles, and this physical cementation is more effective in improving BSCs compressive strength than adhesion solely relying on EPS [68]. Meanwhile, the secreted EPS can efficiently adsorb positively charged nutrient ions such as NH_4_^+^ and K^+^ in the soil through surface functional groups [69,70], forming a dual effect of “biological cementation-nutrient adsorption”: it not only reduces nutrient loss caused by frequent wind-sand activities in arid regions but also significantly improves the bioavailability of surface soil nutrients [3,71]. This characteristic enables it to substantially improve the microhabitat, laying a foundation for the sustained prosperity of itself and associated microorganisms (such as cyanosphere nitrogen-fixing bacteria) and the stable development of BSCs, which perfectly conforms to its role as an “ecosystem engineer” in the early successional stage [72,73].

Gene quantitative analysis provides direct molecular evidence for the above mechanism: Our qPCR data show that the copy numbers of bacterial 16S rRNA, cyanobacterial 16S rRNA, and diazotrophic *nif*H gene in the *M. vaginatus* inoculation groups (SM, SMB) are significantly higher than those in other treatment groups (*p* < 0.05; Figure 2). Among them, the cyanobacterial gene copy number in the *M. vaginatus* groups is at least 5-fold higher than that in the *Leptolyngbya* sp. group, and the *nif*H gene abundance in the SMB group is 3.00-fold that of the SL group and 4.21-fold that of the S control group. This is consistent with the pattern reported in relevant studies of the Horqin Sandy Land: “under grazing disturbance, the abundance of photosynthetic cyanobacteria and the abundance of nitrogen-fixing genes decrease significantly in sync (81.01% decrease in photosynthetic cyanobacteria abundance, 87.72% decrease in nitrogen-fixing gene abundance)” [74]. This confirms that *M. vaginatus* can form a functional synergistic network by constructing a community core.

This evidence supports that *M. vaginatus* can form a functional synergistic network by constructing core microbial taxa of the community. Studies have verified that the polysaccharide sheaths secreted by *M. vaginatus* can bind soil particles, construct stable microhabitats, and retain water and nutrients, thereby providing critical microhabitat support for the proliferation and functional expression of nitrogen-fixing bacteria [75]. In contrast, although the *Leptolyngbya* sp.-inoculated groups could specifically enrich fungi and nitrogen-fixing bacteria, the enriched microorganisms failed to develop into the dominant flora during Cyano-BSCs formation due to the lack of such microhabitat construction capacity. This is highly consistent with the conclusion that the functions of nitrogen-fixing bacteria are susceptible to microhabitat limitations in biocrusts not dominated by *M. vaginatus* [75].

Structural equation model (SEM) analysis further confirmed that the pioneer species *M. vaginatus* CM01 directly regulates the microbial community structure (path coefficient = 0.59, *p* < 0.05) and indirectly drives the optimization of soil environmental factors (path coefficient = 0.64, *p* < 0.05), forming a positive regulatory chain of “cyanobacteria-community-environment”. This regulatory mechanism is highly consistent with the natural successional law of desert Cyano-BSCs, that is, the dominant colonization of *M. vaginatus* creates favorable conditions for the subsequent colonization of microorganisms, promoting the development of BSCs from the primary stage to a more stable mature stage. The results of this study provide theoretical support for the artificial cultivation of Cyano-BSCs in arid regions, clarify the application value of *M. vaginatus* as a core inoculant, and also provide experimental basis for understanding the multi-factor synergistic mechanism of Cyano-BSCs formation.

## 5. Conclusions

This study compared two cyanobacterial species with distinct functional traits to elucidate the core mechanism underlying the formation of artificial Cyano-BSCs. The key finding is that the ability of cyanobacteria to construct physical microhabitats in the early stage acts as a more critical limiting factor than nutrient supply. *M. vaginatus* leverages its filamentous structure and strong habitat adaptability to provide a growth substrate for microorganisms, driving microbial community assembly to shift from stochastic diffusion to deterministic selection. This process facilitates the formation of a synergistic microbial network centered on Cyanobacteria, characterized by high diversity, elevated functional gene abundances, and enriched nitrogen-fixing taxa, which collectively enhance Cyano-BSCs development in terms of coverage, OM content, and Chl*a* concentration. In contrast, *Leptolyngbya* sp., lacking the capacity for physical microhabitat construction, fails to establish a stable colonization environment for microorganisms despite its potential nitrogen-fixing ability. This deficiency results in low microbial diversity, reduced functional gene abundances, and the inability to form an efficient synergistic network, ultimately leading to poor Cyano-BSCs development.

In summary, this study reveals the key determinants of Cyano-BSCs formation from the perspectives of microbial community assembly, gene characteristics, and diversity. It highlights that cyanobacterial species proficient in physical microhabitat construction should be prioritized as pioneer inoculants for artificial Cyano-BSCs restoration. This finding challenges the traditional notion that “nitrogen-fixing cyanobacteria are more conducive to biocrust formation” and provides a theoretical basis and microbial pathway for the development of efficient and targeted Cyano-BSCs cultivation technologies in arid ecosystems.

## Figures and Tables

**Table 1:** Physicochemical properties of Cyano-BSCs under different treatments.

Samples	S	SB	SM	SMB	SL	SLB
Coverage (%)	N.D.	N.D.	23.33 ± 0.58 a	20.67 ± 0.88 a	3.10 ± 0.10 b	1.07 ± 0.07 b
OM (g·kg^−1^)	0.68 ± 0.30 c	0.99 ± 0.31 c	3.36 ± 0.51 a	4.10 ± 0.22 a	1.26 ± 0.29 bc	2.10 ± 0.36 b
Chl*a* (μg·g^−1^)	1.12 ± 0.01 c	1.45 ± 0.01 c	9.64 ± 0.03 b	13.40 ± 0.56 a	9.85 ± 0.04 b	9.85 ± 0.06 b

Note: S, soil control without inoculation; SB, soil inoculated with biocrust leachate alone; SM, soil inoculated with *M. vaginatus* CM01 alone; SMB, soil co-inoculated with *M. vaginatus* CM01 and biocrust leachate; SL, soil inoculated with *Leptolyngbya* sp. CT01 alone; SLB, soil co-inoculated with *Leptolyngbya* sp. CT01 and biocrust leachate. Values are presented as mean ± standard deviation (*n* = 5). Letters (a, b, c) in superscript indicate significant differences among groups based on Duncan’s multiple range test (*α* = 0.05, *p* < 0.05). N.D. = not detected.

**Table 2:** Topological properties of the molecular ecological networks for the bacterial communities in Cyano-BSCs.

Network Indexes	S	SM	SL	SB	SMB	SLB
edges	3698	5536	4127	7425	8497	8999
nodes	263	319	312	305	267	225
Positive links (%)	55.19	89.74	67.92	57.87	72.80	51.46
Negative links (%)	44.81	10.26	32.08	42.13	27.20	48.54
R squared value of power law	0.64	0.00	0.00	0.00	0.45	0.84
Average degree (avgK)	20.89	34.71	26.46	40.33	63.60	63.24
Average clustering coefficient (avgCC)	0.48	0.55	0.52	0.44	0.66	0.76
Average path distance (GD)	2.50	2.38	3.04	1.81	2.01	1.78
Geodesic efficiency (E)	0.38	0.42	0.33	0.55	0.36	0.28
Harmonic geodesic distance (HD)	2.52	2.38	3.04	2.33	3.64	2.28
Density (D)	0.11	0.11	0.09	0.20	0.24	0.36
Connectedness (Con)	1.00	1.00	1.00	1.00	1.00	1.00
Relative modularity (RM)	0.56	0.44	0.54	0.16	0.34	0.68

Note: S, soil control without inoculation; SB, soil inoculated with biocrust leachate alone; SM, soil inoculated with *M. vaginatus* CM01 alone; SMB, soil co-inoculated with *M. vaginatus* CM01 and biocrust leachate; SL, soil inoculated with *Leptolyngbya* sp. CT01 alone; SLB, soil co-inoculated with *Leptolyngbya* sp. CT01 and biocrust leachate. Values are presented as mean ± standard deviation (*n* = 5).

## Data Availability

The sequencing data generated in this study have been deposited in the NCBI Sequence Read Archive (SRA) under the BioProject accession number PRJNA1370247. These data are publicly accessible from https://www.ncbi.nlm.nih.gov/bioproject/PRJNA1370247 (accessed on 20 January 2026).

## Supplementary Materials

The following supporting information can be downloaded at: https://www.mdpi.com/article/10.3390/microorganisms14030542/s1, Table S1: Coordinates of Sampling Locations in May 2023.
