# Supplementary material for: The Importance of Microcoleus vaginatus in Shaping Bacterial Communities Essential for the Development of Cyanobacterial Biological Soil Crusts
Source: Microorganisms. 2026 Feb 27;14(3):542. doi: 10.3390/microorganisms14030542 (PMC13028718; doi:10.3390/microorganisms14030542)
Supplement: Supplementary file 1 [file microorganisms-14-00542-s001.zip › microorganisms-4100868-supplementary.pdf]

Table S1. Coordinates of Sampling Locations in May 2023

**Region**	**longitude**	**latitude**
Badain Jaran Desert	100°56′14.7″ E	39°17′25.9″ N
Tengger Desert	103°16′29.0″ E	38°15′43.2″ N
Ulan Buh Desert	106°50′54.9″ E	40°24′30.2″ N
Kubuqi Deser	107°08′42.2″ E	40°13′49.4″ N
Mu Us Sandy Land	107°04′17.3″ E	38°12′37.4″ N
Hunshandak Sandy Land	116°48′44.8″ E	42°12′49.4″ N
Horqin Sandy Land	120°05′23.3″ E	43°31′57.6″ N
Hulunbuir Sandy Land	118°56′12.2″ E	49°15′14.4″ N
